# *Origanum majorana* Ethanolic Extract Promotes Colorectal Cancer Cell Death by Triggering Abortive Autophagy and Activation of the Extrinsic Apoptotic Pathway

**DOI:** 10.3389/fonc.2019.00795

**Published:** 2019-08-21

**Authors:** Nehla Benhalilou, Halima Alsamri, Aysha Alneyadi, Khawlah Athamneh, Asma Alrashedi, Nedaa Altamimi, Yusra Al Dhaheri, Ali H. Eid, Rabah Iratni

**Affiliations:** ^1^Department of Biology, College of Science, United Arab Emirates University, Abu Dhabi, United Arab Emirates; ^2^Department of Pharmacology and Toxicology, Faculty of Medicine, American University of Beirut, Beirut, Lebanon

**Keywords:** colon cancer, abortive autophagy, apoptosis, DNA damage, *Origanum majorana*, HPLC-MS

## Abstract

Colorectal cancer is considered as the third leading cause of cancer death. In the present study, we investigated the potential anticancer effect and the molecular mechanism of *Origanum majorana* ethanolic extract (OME) against human colorectal cancer cells. We showed that OME exhibited strong anti-proliferative activity in a concentration- and time-dependent manner against two human colorectal cancer cell lines (HT-29 and Caco-2). OME inhibited cell viability, colony growth and induced mitotic arrest of HT-29 cells. Also, OME induced DNA damage, triggered abortive autophagy and activated a caspase 3 and 7-dependent extrinsic apoptotic pathway, most likely through activation of the TNFα pathway. Time-course analysis revealed that DNA damage occurred concomitantly with abortive autophagy after 4 h post-OME treatment while apoptosis was activated only 24 h later. Blockade of autophagy initiation, by 3-methyladenine, partially rescued OME-induced cell death. Cell viability arose from 37% in control group to 67% in group pre-treated with 3-MA before addition of OME. Inhibition of apoptosis, however, had a minimal effect on cell viability; it rose from 37% in control group to 43% in group pre-treated with Z-VAD-FMK. We also found that OME downregulated survivin in HT-29 cells. Our findings provide a strong evidence that *O. majorana* extract possesses strong anti-colon cancer potential, at least, through induction of autophagy and apoptosis. These finding provide the basis for therapeutic potential of *O. majorana* in the treatment of colon cancer.

## Introduction

With an approximate of 1.8 million cases globally, and 862,000 documented deaths, colorectal cancer accounts for the third most common cancer worldwide both in men and women (1). Although conventional cancer therapies such as surgery, radiation, and chemotherapy achieve great advancements in cancer treatment and management, the undesired side effects that are accompanied by such treatments deleteriously affect the health of the patients (2). Therefore, the quest for alternative therapies with less toxicity and more potent anti-cancer drug are needed.

Currently, many researches in the field of cancer therapy, are being focused on plants as valuable source for identification and development of new anti-cancer agents for cancer treatment as they possess anti-tumor properties with minimal or no toxicity (3). One of the plants that gained a lot of interest is *Origanum majorana* L. (OM), commonly known as marjoram. OM is an herbaceous plant that belongs to the family of Lamiaceae, mainly distributed in the Mediterranean region and can grow up to 60 cm. The usage of OM for flavor and aroma dates back to ancient times. Traditionally, the leaves of OM are used for its medicinal properties to cure insomnia, asthma, gastritis and nervousness (4). Several studies showed that OM extract exhibited an anti-microbial activity (5), inhibited platelet adhesion, aggregation and secretion (6), attenuated nephrotoxicity of cisplatin anti-cancer drug (7), showed positive effects in acute infectious diarrhea (8), decreased the incidence of ulcers and replenished the depleted gastric wall mucus (9). Our group has previously shown that OME exhibits a potent inhibitory activity against triple negative breast cancer (TNBC). We showed that OME promoted mitotic arrest, induced apoptosis as well as inhibited migration, metastasis and tumor growth of TNBC (10, 11).

The aim of the current study is to investigate the cytotoxic effect of OME against human colorectal cancer cells. Our results revealed that OME exerts a cytotoxic effect on colon cancer cells by inducing mitotic arrest and activating of autophagic and apoptotic cell death.

## Materials and Methods

### Cell Culture, Chemicals, and Antibodies

Human colon cancer cells HT-29 (Cat# 300215) and CaCo-2 (Cat # 300137) were purchased from CLS (cell lines service, Germany). Cells were cultured in DMEM supplemented with 10% fetal bovine serum and 100 U/mL penicillin/streptomycin at 5% CO_2_, 37°C and 95% humidity. 3-methyladenine (3-MA) and Z-VAD-FMK were obtained from sigma-Aldrich. Antibodies against target proteins used in this study are: caspase 8, caspase 7, LC3 and Beclin-1 (Cell Signaling, USA); cleaved caspase 3, Cyclin B1, H3 phospho-Ser10, γH2AX (Millipore), TNFα, p62/SQSTMI and cleaved PARP (Abcam), survivin and β-actin (Santa Cruz Biotechnology).

### Preparation of *Origanum majorana* Ethanolic Extract (OME)

The plant was collected from a private commercial farm located at 33° 16′ 54′′ N and 35° 14′ 51′′ E. The farm is located in Tire region, Lebanon and the approval of the owner was obtained before collecting the fruit or commencing any experiments. This plant is neither endangered nor protected by any laws and it is readily and commercially available in the market. *Origanum majorana* plant, at the time of collection, was identified by Dr. Ali Al-Khatib, a plant biologist at the Lebanese International University (Lebanon). The dried leaves, used for the extraction, were further identified and confirmed by Dr. Mohamed Tahar Moussa, plant taxonomist at the United Arab Emirates University where a voucher specimen of the plant (No. 14670) was deposited at the National Herbarium, College of Science, Department of Biology, United Arab Emirates University.

*Origanum marjorana* ethanolic extract (OME) was prepared as previously described (10). Briefly, dried leaves powder (5.0 g) was extracted in 100 mL of 70% absolute ethanol and the mixture was kept in the dark for 72 h in a refrigerator without stirring. Afterward, the mixture was filtered, and the filtrate was evaporated to dryness using a rotary evaporator at room temperature. The green residue was kept under vacuum for 2–3 h and its mass was recorded. The residue was stored at −20°C until further use.

### HPLC-MS Identification of Constituents in *Origanum majorana* Ethanolic Extract

The identity of *O. majorana* was analyzed by LC-MS (6420 Triple Quadrupole, Agilent Technologies). Sample of *O. majorana* ethanolic extract was filtered using 0.45 μm syringe filter preceding the analyses. The instrument was fitted with a Agilent EclipsePlus-C18 column (1.8 μm particle size, 2.1 × 50 mm length, Agilent Technologies, USA) maintained at 35°C, coupled to a tunable UV-Vis detector (Agilent Technologies, USA) and 6420 Triple Quadrupole LC/MS System (Agilent Technologies, USA). The mobile phases used were A = 0.1% formic acid and B = acetonitrile, and the gradient was: 0–2.5 min: 0% B, 2.5–15 min: 20–100% B, 15–18 min: 100% B, and 18–25 min: 5% B with 0.2 ml/min. Electrospray ionization (ESI) source was used in LC-MS system in positive polarity. LC-MS operating conditions were as follows: capillary voltage: 4 kV; the nebulizer pressure was 45 psi; drying gas flow was 11 L/min and drying temperature was 325°C. The mass range monitored was from 100 to 1,000 Da. Compounds were identified based on their Molecular weight (MW) and retention time (RT).

**Figure 1:**
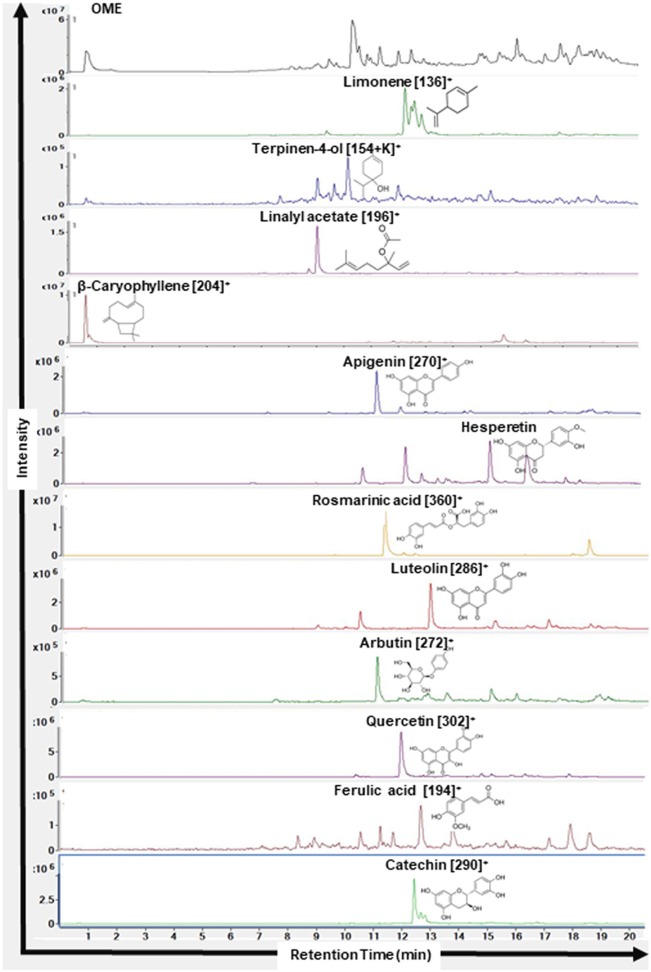
Chromatograms of compounds identified in ethanolic extract of *Origanum majorana* L. by HPLC-MS. Name of the compounds, their respective chemical structures, molecular masses, and retention times are shown.

As shown in Figure 1, HPLC/MS analysis revealed the presence of several peaks which indicated the presence of 12 compounds [Limonene (MW: 136 g/mol, RT: 12.2 min), Terpinen-4-ol (MW: 153 g/mol, RT: 10.2 min), Linalyl acetate (MW: 196 g/mol, RT: 12.2 min), β-Caryophyllene (MW: 204 g/mol, RT: 10.6 min), Apigenin (MW: 270 g/mol, RT: 11.1 min), Hesperetin (MW: 302 g/mol, RT: 15.2 min), Rosmarinic acid (MW: 360 g/mol, RT: 11.3 min), Luteolin (MW: 286 g/mol, RT: 12.9 min), Arbutin (MW: 272 g/mol, RT: 15.9), Quercetin (MW: 302 g/mol, RT: 12.6 min), Ferulic acid (MW: 194 g/mol, RT: 12.7 min), Catechin (MW: 290 g/mol, RT: 12.4 min)].

### Measurement of Cellular Viability

Cells (7 × 10^3^ cells/well) were seeded in triplicates in 96-well culture plates. After overnight incubation, cells were treated with various concentrations of OME or vehicle for another 24 and 48 h. At the end of incubation, cell viability was measured with the Cell cytotoxicity assay kit (Abcam) following the manufacturer's instructions.

Cell viability was also measured by counting live cells using the Muse Count and Viability Kit (Millipore) as previously described (12).

### Colony Formation Assay

For anchorage-dependent colony formation assay, HT-29 cells were seeded (2 × 10^3^ cells/well) in 6-well culture plates, and maintained for 10 days at 37°C to form colonies. At day 10, formed colonies were treated with various concentrations of OME and were maintained at 37°C for 5 additional days. At day 15, plates were washed 3 times with PBS, fixed for 15 min with 4% formalin and stained with 0.01% crystal violet for 30 min. The colony number was counted and their surface area in each well was measured using the imageJ software.

### Cell Cycle Analysis

Briefly, HT-29 cells were exposed to different concentrations of OME or vehicle. After 24 h incubation, cells were harvested, washed with 1X PBS and then fixed with ice-cold 70% ethanol and stored at −20°C overnight. Cell cycle analysis was performed using the Muse™ Cell Analyzer (Millipore) as previously described (13). Analysis of DNA content of cells at different phases of cell cycle was determined using the FlowJo software.

### Whole Cell Extract and Western Blotting Analysis

HT-29 cells were seeded in 100 mm culture dishes at a density of 3 × 10^6^ and cultured for 24 h. After OME treatment, protein extraction and Western blotting were performed as previously described (12). All Western blots shown are representative of three independent experiments.

### Statistical Analysis

Statistical analyses were carried out using SPSS version 21 software. Data were presented as group mean ± SD. The data were analyzed via one-way ANOVA followed by LSD's *post-hoc* multiple comparison test. A *p* < 0.05 was considered statistically significant.

## Results

### *Origanum majorana* Extract Inhibits Cellular Viability of Human Colorectal Cancer Cells

**Figure 2:**
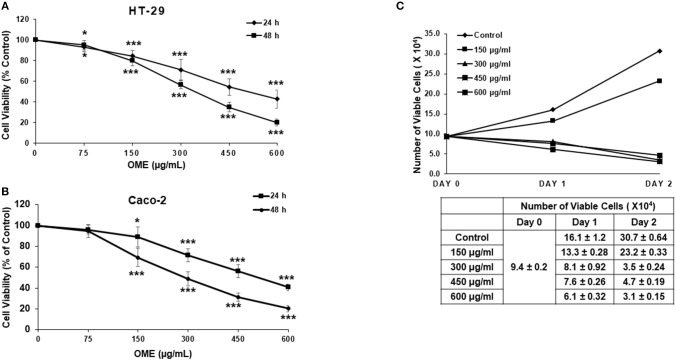
*Origanum majorana* ethanolic extract inhibits cellular viability of colorectal cancer cells. **(A)** Exponentially growing HT-29 and **(B)** Caco-2 colon cancer cells were treated with and without of various concentration (0, 150, 300, 450, and 600 μg/mL) OME for 24 and 48 h. Viability was measured using a colorimetric assay as described in section Materials and Methods. Values are represented as mean ± SD of *n* = 4 (*p < 0.05 and ***p < 0.001). **(C)** HT-29 cells were exposed to OME for 24 and 48 h and number of viable cells, using a fluorescent dye, was monitored as described in section Materials and Methods using the Muse Cell Analyzer (Millipore). Data represent the mean ± SD of *n* = 3 carried out in triplicate.

We have examined the anti-proliferation effect of OME on two human colorectal cancer cell lines (HT-29 and Caco-2 cells). Cells were treated with increased concentrations of OME for 24 and 48 h. In both cell lines, OME caused significant growth inhibition, in a concentration and time-dependent manner as shown in Figures 2A,B. IC50 values are 498 and 342 μg/mL at 24 and 48 h for HT-29 and 506 and 296 μg/mL at 24 and 48 h for Caco-2 cells. It is worth mentioning that there is no color interference between the extract and the reagent used to measure cellular viability.

Cell viability was also measured by counting live cells using the Muse cell counting kit, which differentially stains viable and dead cells. Results showed a concentration of 150 μg/mL of OME, reduced cellular proliferation of HT-29 when compared to control cells. However, concentrations of 300, 450, and 600 μg/mL of OME caused time-dependent decline in the number of viable cells when compared to the number of cells counted at the day of treatment (day 0) (Figure 2C) hence, indicating cell death.

### *Origanum majorana* Extract Inhibits HT-29 Colony Growth

**Figure 3:**
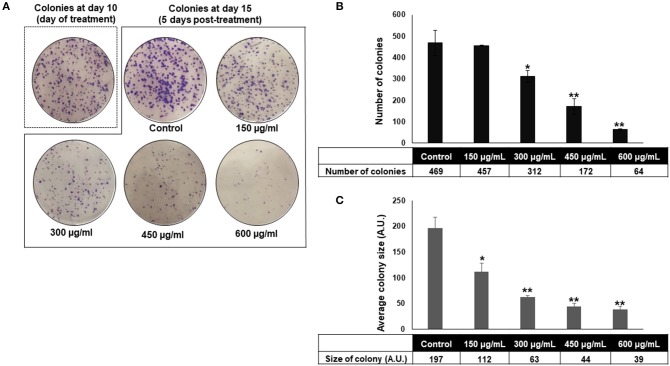
*Origanum majorana* inhibits HT-29 colony growth. **(A–C)** Inhibition of formed HT-29 colony growth by various concentrations of OME (0, 150, 300, 450, and 600 μg/mL) was assessed by measuring the number and average size (surface area) of the colonies obtained in control and OME-treated plate as described in section Materials and methods. Values are represented as mean ± SD of *n* = 3 (*p < 0.05 and **p < 0.005).

The anti-colon cancer effect of OME was further examined by testing the ability of OME to modulate the proliferative capacity of HT-29 to form colonies. After HT-29 cells were allowed to form colonies for 10 days, wells were replaced with fresh media with or without OME and colonies were let to grow for 5 additional days. Results showed that OME caused a significant decrease in concentration-dependent manner, not only, in size (Figure 3C) but also in number (Figure 3B) of the already formed colonies and thus, clearly indicating massive cell death of HT-29 cells (Figures 3A,B).

### *Origanum majorana* Extract Induces a Mitotic Arrest

**Figure 4:**
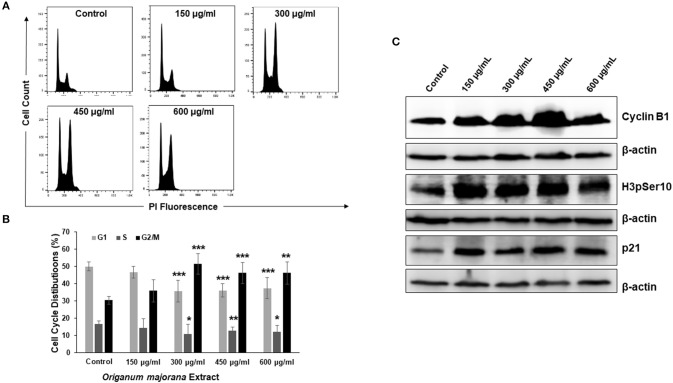
OME induces a mitotic arrest in HT-29 cells. **(A,B)** Cell cycle distribution analysis in HT-29 cells treated with and without OME (0, 150, 300, 450, and 600 μg/mL) for 24 h. Values are represented as mean ± SD of *n* = 3 (*p < 0.05, **p < 0.005, and ***p < 0.001). **(C)** Alteration in proteins associated with cell cycle regulation in OME-treated HT-29 cells.

In order to investigate the mechanism through which OME exerts its cytotoxic and colony growth inhibition effect on HT-29 cells, cell cycle analysis was performed. We found that a concentration of 300, 450, and 600 μg/mL of OME caused a significant inhibition of cell cycle progression at G2/M. Indeed, the population arrested cells at G2/M cells arose from 30% in control to 51% in OME-treated cells (Figures 4A,B). To determine at which stage (M or G2) cell cycle arrest occurred, we examined the phosphorylation status of histone H3, a marker of mitosis. OME at concentrations of 300, 450, and 600 μg/mL significantly increased the level of H3p(Ser10) (Figure 4C). The level of cyclin B1, whose upregulation invokes mitotic arrest, was also examined and was found to be upregulated as well (Figure 4C). We also found that OME induces a concentration dependent increase of the cell cycle regulator p21 (Figure 4C), hence suggesting that p21 upregulation may contribute to the mitotic arrest induced by OME.

### *Origanum majorana* Extract Activates Caspase 3 and Caspase 7-Dependent Extrinsic Apoptotic Pathway

**Figure 5:**
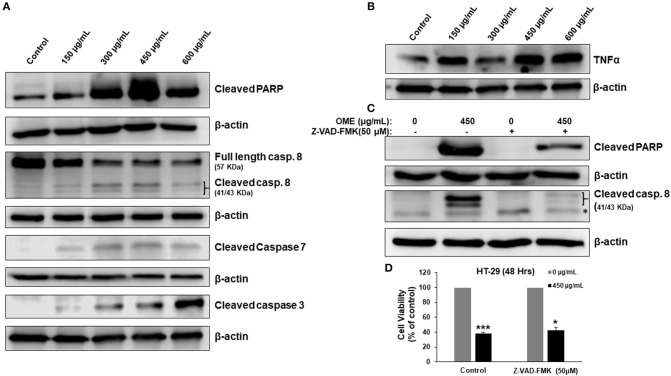
Activation of extrinsic apoptotic pathway and upregulation of TNF-α in OME-treated HT-29 cells. **(A)** Western blot analysis of caspase 3, 7, and 8 activation and PARP cleavage in HT-29 cells. Cells were treated with or without increasing concentration (0, 150, 300, 450, and 600 μg/mL) of OME for 48 h, then whole cell proteins were extracted and subjected to Western blot analysis for the markers of apoptosis **(B)** Western blot analysis of TNF-α **(C)** Western blot analysis of cleaved PARP in cells pretreated for 1 h with and without Z-VAD-FMK (50 μM) followed by treatment with OME (450 μg/mL) for 48 h. **(D)** Inhibition of apoptosis has a minimal effect of OME-induced cell death. HT-29 cells were pretreated with Z-VAD-FMK as described above and then treated for 48 h with 450 μg/mL OME. Cell viability was determined as described in section Material and Methods. Values are represented as mean ± SD of *n* = 3 (*p < 0.05 and ***p < 0.001).

We have shown that OME induced a concentration-dependent cell death of HT-29 (Figure 2C). Therefore, we examined whether this cell death was associated with the activation of apoptosis. Western blotting analysis showed an accumulation of cleaved PARP, active caspase 8, 3, and 7 in response to OME (Figure 5A). Interestingly, we found that TNF-α, a cytokine involved in the initiation of the extrinsic apoptotic pathway, was also upregulated by OME (Figure 5B) suggesting that OME activates the extrinsic apoptotic pathway through activation of the TNF-α signaling pathway.

Next, we examined whether apoptosis is the sole mechanism of cell death activated by OME. Blockade of apoptosis by the pancaspase inhibitor, Z-VAD-FMK (50 μM), revealed by the absence of cleaved PARP and cleaved caspase 8 (Figure 5C), led to a minimal recovery of cell viability. Cell viability increased from 37% in control cell treated with OME only to 43% when cells were pre-treated with Z-VAD-FMK (Figure 5D), suggesting that apoptosis is not the main way through which cell dies and that another mechanism of cell death might be activated as well.

### *Origanum majorana* Extract Induces Abortive Autophagy in Colon Cancer Cells

**Figure 6:**
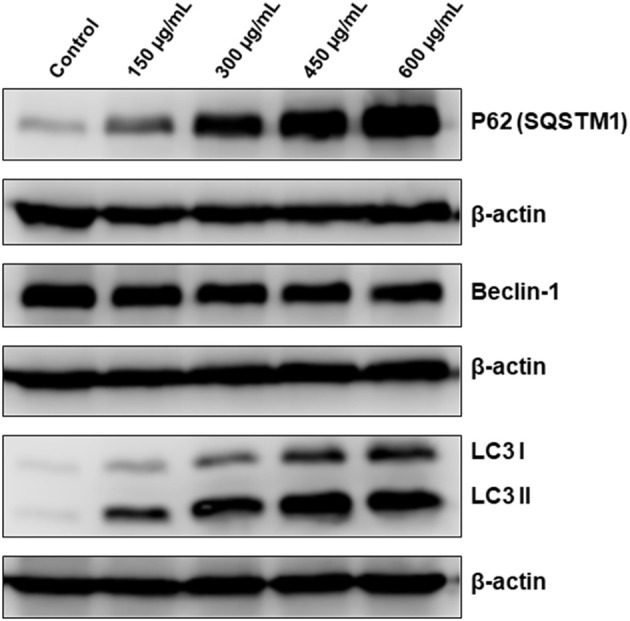
OME induces abortive autophagy in HT-29 cells. Western blotting analysis of LC3II, p62(SQSTM1), and Beclin-1 expression OME-treated HT-29 cells. Cells were treated with or without increasing concentration (0, 150, 300, 450, and 600 μg/mL) of OME for 48 h, then whole cell proteins were extracted and subjected to Western blot analysis, as described in section Materials and Methods, for LC3II, 62(SQSTM1), and Beclin-1.

Having shown that apoptosis is not the sole mechanism of cell death activated by OME, we interrogated whether autophagy is activated by OME in colon cancer cells. We found that that OME caused an increase in the accumulation of lipidized LC3II, a marker of autophagy, in a concentration-dependent manner, suggesting that autophagy is occurring or at least initiated in HT-29 cells (Figure 6). Next, we examined the expression of Beclin-1, an autophagy effector playing a key role in autophagosome. Western blot analysis showed that the level of Beclin-1 slightly decreased in OME-treated cells compared to control cells (Figure 6) and hence, suggesting that autophagy is occurring through Beclin-1-independent mechanism. Further, we scored for p62(SQSTM1), a ubiquitin-binding protein, a marker of autophagic flux degraded in productive autophagy. Strikingly, we found that level of p62(SQSTM1 increased dramatically in concentration-dependent and sustained manner (Figure 6). It is well-documented that p62 is preferentially degraded during autophagy; however, its level remains unchanged or increased during abortive autophagy. Hence our results suggest that autophagy flux was disrupted leading to abortive autophagy in HT-29 cells.

### *Origanum majorana* Extract Induces DNA Damage in Colon Cancer Cells

**Figure 7:**
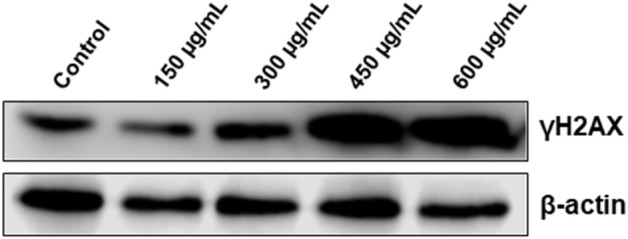
OME induces DNA damage in response to OME treatment in HT-29 cells. HT-29 cells were treated with increasing concentrations (0, 150, 300, 450, and 600 μg/mL) of OME for 48 h. DNA damage was examined by western blotting by measuring the level of phosphorylated H2AX.

It is well-established that DNA damage can trigger cell cycle arrest, autophagy and apoptosis in cancer cells. Because the three events were observed in OME-treated cells, we decided to investigate whether OME exert its anti-colon cancer affect through induction of DNA damage. We found that OME treatment caused an increase in the levels of γH2AX (Figure 7), indicative of double strand breaks.

### Abortive Autophagy, and DNA Damage Precedes Apoptosis Activation

**Figure 8:**
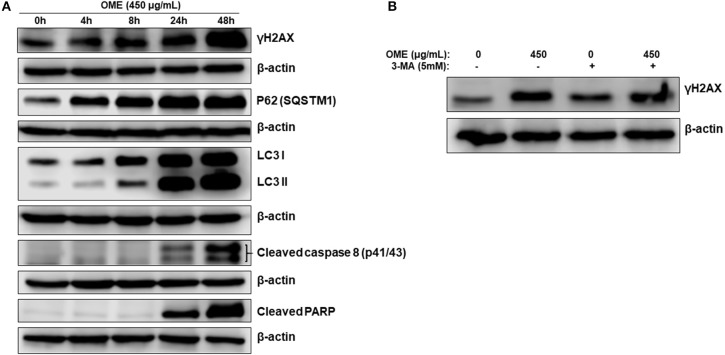
DNA damage and autophagy precedes apoptosis in OME-treated HT-29 cells. **(A)** Time-course analysis, by Western blotting, of PARP and caspase 8 cleavage, LC3-II, p62 (SQSTM1), γH2AX, and H3pser10 accumulation in OME-treated HT-29 cells. Cells were treated with 450 μg/mL OME and proteins were extracted at the indicated time-points (0, 4, 8, 24, and 48 h) as described in section Materials and Methods. **(B)** Western blot analysis of γH2AX accumulation in HT-29 cells pre-treated with 3MA. Cells were pretreated with or without 3-MA (5 mM) for 1 h and then OME (450 μg/mL) was added, and cells were incubated for 48 h.

We next monitored the accumulation of markers of DNA damage (γH2AX), autophagy (LC3II and p62) and apoptosis (cleaved caspase 8 and cleaved PARP) over time. We found that lipidized LC3II, upregulation of p62 and accumulation of DNA damage were detected concomitantly as early as 4 h post-OME treatment (Figure 8A). Again, LC3II and p62 showed sustained accumulation over time, further confirming the occurrence of abortive autophagy. Apoptosis, however, occurred only 24 h post-treatment and was maximal at 48 h. These results suggest that DNA damage and autophagy are the earliest consequence of OME-treatment to HT-29 cells.

To determine which event (DNA damage or autophagy) occurred first, we measured the level of γH2AX in HT-29 cells pre-treated with 3-MA (5 mM), an autophagy inhibitor of autophagosome formation, and then treated with OME. We found that the inhibition of autophagy initiation did not prevent DNA damage induced by OME (Figure 8B), hence, suggesting that DNA damage preceded autophagy activation.

### Inhibition of Autophagy Initiation Partially Reduced Cell Death Induced by OME and Apoptosis Activation Depend on the Induction of Autophagy

**Figure 9:**
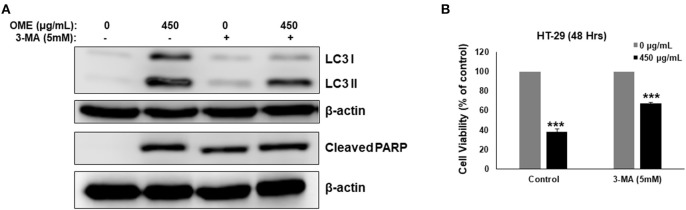
Inhibition of autophagy decreases OME-induced cell death in HT-29 cells. **(A)** Analysis of LC3-II and cleaved PARP accumulation in HT-29 cells pre-treated with 3-MA. Cells were pretreated with or without 3-MA (5 mM) for 1 h and then OME (450 μg/mL) was added, and cells were incubated for 48 h. **(B)** Inhibition of autophagy reduces cell death induced by OME. HT-29 cells were pretreated with 3-MA for 1 h and then for 48 h with 450 μg/mL OME. Cell viability was determined as described in Material and Methods. Values are represented as mean ± SD of *n* = 3 (***p < 0.001).

We have shown that OME induced abortive autophagy as early as 4 h post-treatment while apoptosis was activated only at 24 h. The contribution of autophagy in OME-induced cell death was, therefore, examined in cells pre-treated with 3-MA (5 mM). Blockade of autophagy by 3-MA was confirmed by the observed decrease in the conversion of LC3-I to LC3-II (Figure 9A). Interestingly, cell viability markedly increased when autophagy was blocked compared with control group treated with OME only (Figure 9B). Indeed, cell viability arose from 38% in control group to 67% in group pre-treated with 3-MA before addition of OME (Figure 9B). It is noteworthy to mention that the blockade of autophagy reduced the level of cleaved PARP in HT-29 cells (Figure 9A); hence suggesting that activation of apoptosis is somehow dependent on the initiation of autophagy.

### *Origanum majorana* Downregulates the Level of Survivin

**Figure 10:**
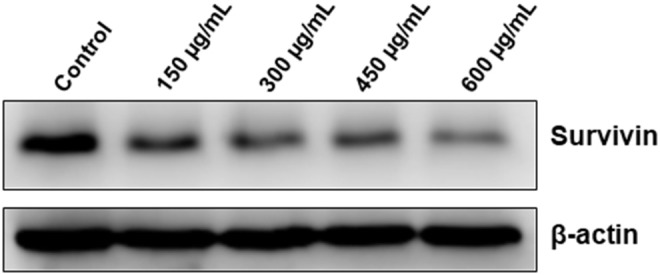
Downregulation of survivin by OME in HT-29 cells. HT-29 cells were treated with increasing concentrations (0, 150, 300, 450, and 600 μg/mL) of OME for 48 h and the level of survivin was assessed by Western blotting.

In addition to its role in mitosis and apoptosis, survivin might also protect cell from death through a mechanism involving autophagy. We found that OME significantly downregulated the level of survivin in HT-29 cells (Figure 10). This result suggests that downregulation of survivin by OME could account, although may be not solely, in sensitization of HT-29 cells to autophagic and apoptotic cell death. It is noteworthy to mention that emerging evidences suggests that survivin can also repress autophagy, it is then tempting to think that downregulation of survivin could contribute, at least partly, in promoting sustained autophagy in response to OME.

## Discussion

We have previously shown that *O. majorana* extract exerts an anti-proliferative, anti-metastatic and anti-tumor growth against the highly proliferative and invasive TNBC. We showed that OME induced mitotic arrest, DNA damage and triggered extrinsic apoptotic pathway (10). We also showed that OME inhibited tumor growth of MDA-MB-231 *in ovo* (11). Other studies reported that OM inhibited the viability of human hepatocarcinoma HepG2 cells and inhibited the NFkB activity (14). Here we extended our study by examining the activity of OME against colorectal cancer. We report for the first time the anti-colorectal cancer activity of OME. Our findings demonstrate that OME reduces the viability, inhibits colony growth, induces mitotic arrest and DNA damage in colon cancer cells. In addition, we show that OME triggered abortive autophagy and activated a caspase 3 and 7-dependent extrinsic apoptotic pathway, most likely through mechanism involving TNFα pathway. Moreover, we also show that OME downregulated survivin.

Autophagy is a highly conserved lysosomal catabolic process by which damaged cellular organelles and misfolded proteins are degraded under stress conditions (15). Increasing evidence supports the notion that inhibition of autophagy enhances the efficacy of chemotherapeutic agents and hence can be used as anticancer therapeutic strategy (16). To date, a large number of anticancer agents were shown to elicit abortive autophagy in cancer cells. For example, salinomycin induced ROS-dependent abortive autophagy associated with necrotic cell death in glioblastoma. Interestingly, alleviation of ROS production restored the autophaic flux, hence suggesting that an oxidative stress could play a role in blocking the autophagy flux (17). Also, lovastatin and farnesyl transferase inhibitor (FTI)-1 co-treatment was shown to inhibit the completion of autophagy program and induce non-apoptotic cell death in the human malignant peripheral nerve sheath tumor cell line STS-26T (18). The exact mechanism through which lovastatin and FTI-1 combination induces abortive autophagy is still unknown. A possible mechanism regulating abortive autophagy has been postulated by Clearhout and collaborators. In this study, the authors showed that a productive autophagy requires the coatomer complex I (COPI) and they showed that the reduction of its members decrease cell survival and induce abortive autophagy in cancer cells (19). Here, we show for the first time that OME treatment resulted in substantial and sustained accumulation of LC3II and p62, suggestive of abortive autophagy, in HT-29 cells associated with cell death. Interestingly, and in contrast to the previous studies which reported a non-apoptotic cell death in cancer cells undergoing abortive autophagy (17, 18), OME seems to activate autophagic and apoptotic cell death. Further investigations are needed to dissect how OME disrupts autophagy flux and triggers both cell death programs.

Although apoptosis and autophagy represent two different mechanisms of programed cell death, PCD I and PCD II, respectively, a cross-talk between the two mechanisms exists. Still, the intricate interaction between these two mechanisms is still controversial in cancer treatment. Autophagy appears to play a dual role in cancer cell. It plays a protective role in helping cancer cells to survive by escaping from apoptosis (20). whereas, under different conditions, autophagy can stimulate apoptosis in cancer cells. Interestingly, under some situations, it was reported that apoptosis and autophagy can exert synergetic effects, whereas in other conditions autophagy can be initiated only when apoptosis is inhibited (20). Therefore, what determine the role of autophagy in cancer cells seems to depend upon the cell type, nature and duration of stimulus (21). In our study, we showed that cell death occurs mainly through autophagy mechanism possibly as result of excessive abortive autophagy. Although apoptosis was activated upon OME treatment, it does not seem to define the main mechanism of cell death. The following experimental evidences supports this hypothesis. Our results showed that activation of autophagy occurred as early as 4 h post-OME treatment, while activation of apoptotic pathway occurred only after 24 h pointing out that autophagy preceded apoptosis process. In addition, blockade of autophagy initiation by 3-MA markedly reduced cell death, while inhibition of apoptosis by Z-VAD-FMK had only a slight effect on cellular viability. Based on these findings, we have hypothesized that PCDII represents the main mechanism of cell death in response to OME, and the minimal observed apoptosis arises as secondary response due to extensive cellular damage resulting from continuous exposure of HT-29 cells to OME. Indeed, our claim is supported by the fact that prolonged OME treatment led to dramatic increase in DNA damage (Figures 7, 8A).

DNA damage elicits various cellular responses including cell cycle arrest and execution of programmed cell death through different molecular mechanisms (22). What defines these distinct death fates in response to genotoxic stress still remain unclear. It is reasonable to think that the magnitude of DNA damage determines the response of stressed cells. Interestingly, here we found that OME induced concentration-dependent DNA damage, that occurred upstream of autophagy induction and activation of apoptosis. This result is in agreement with our previous findings in breast cancer where we showed that OME exerted a genotoxic effect on MDA-MB-231 cells (10). We believe that prolonged exposure of colon cancer cells to high concentration of OME causes overwhelming amount of DNA damage, which inevitably resulted in prolonged (abortive) autophagy followed by apoptosis with both events contributing to cell death.

Inhibitor of apoptosis proteins (IAPs), which includes survivin, represents a family of anti-apoptotic proteins that bind and inactivate active caspases (23–25). The expression level of survivin protein was found to be upregulated in several cancer types (26) and correlated with tumor progression and associated with increased resistance to chemotherapy. Thus, survivin protein represents an attractive target in cancer therapy. Indeed, downregulation of survivin by chemotherapeutic agents or RNA interference resulted in caspase activation and increased apoptotic cell death in cancer cells (27, 28). In addition to its role in promoting cell survival through inhibition of apoptosis, it has been postulated that survivin also plays a role as mitotic inducer. Dai et al. showed that depletion of survivin in HepG2 caused a G2/M arrest followed by apoptosis (29). Increasing evidences suggests that survivin protein, in addition to its role in mitosis and apoptosis, may also protect cell from death through a mechanism involving autophagy. Indeed, inhibition of survivin by the small-molecule drug YM155, survivin suppressant, causes significant autophagy-dependent cell death in adenoid cystic carcinoma (30). Very recently, Humphry et al. showed that survivin function in a pro-survival manner by inhibiting excessive autophagy in cancer cells (31). The authors showed that cells expressing survivin accumulated p62 significantly more slowly than control cells (31). Interestingly, here we showed that survivin was severely depleted by OME in HT-29 cells. It becomes then tempting to postulate that downregulation of survivin by OME could account, at least in part, in the cell cycle arrest, excessive autophagy, and apoptosis in colon cancer cells. Further investigation are underway to elucidate the mechanism through which OME downregulates survivin and its contribution in cell death.

Phytochemical analysis carried out by several groups including this study revealed that OME is rich in bioactive compounds possessing anticancer activity (32–35). Indeed, one of the major constituents present OME is luteolin, a dietary flavonoid able to decrease the viability of various cancers cells including lung, colon, liver, and breast cancer cells (36). Luteolin was able to trigger the intrinsic as well as the extrinsic apoptotic pathways in a variety of human cancer cells (37). In addition, luteolin as shown to trigger autophagy in the Metastatic Squamous Cell Carcinoma Cells (38) and in Hepatocellular Carcinoma (39). β-Caryophyllene, also present in OME, is emerging as natural compound with anticancer potential. β-Caryophyllene was reported to inhibit cellular viability of various cancer cell lines including the HCT116 colon cancer cells (40). Quercetin, another abundant compound in OME, was also reported to possess anticancer potential *in vitro* and *in vivo* against various types of cancer (41). Quercetin was reported to induce G2/M arrest and promotes autophagic cell death through ERK activation in SW620 and HCT116 colon cancer cells (42). Rosmarinic acid, another abundant compound in OME, was shown to induce G0/G1 arrest, triggers apoptosis and inhibits migration and invasion of HCT116 and CT26 colorectal cancer cells (43). It appears then that the presence of these bioactive compounds in *O. majorana* extract may contribute to its anticancer activity.

Safety wise, OM did not show adverse effects on humans and animals and thus can be considered as relatively safe. It is noteworthy to mention that we have previously shown that concentrations of OME up to 450 μg/mL is perfectly safe on chick embryo in an *in ovo assay* (11). However, we believe that if the concentration goes significantly higher than 600 μg/mL this might impart deleterious effect on the viability not only on cancer but also on normal cells. Makrane et al. showed that OM exhibited no sign of toxicity in mice fed for 14 days with doses of up to 10 g/kg of aqueous extract of OM (8). They showed that all treated mice survived being active and healthy during the period of treatment. These authors predicted that LD_50_ of OM will be higher than 10 g/kg. Based on our previous data and the above cited report, we can conclude that the concentrations used in the present study are lower than the one shown to be safe in animal models. Finally, FDA has given “Generally Recognized As Safe” status for *O. major*ana.

**Figure 11:**
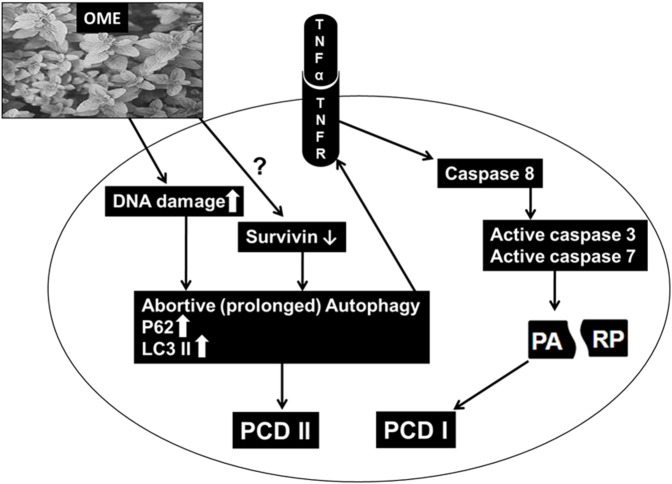
Hypothetic model of OME on colon cancer cells.

In summary, our findings are consistent with the hypothetic model (Figure 11) showing the possible mechanism of action through which OME exerts an anti-proliferative effect against colorectal cancer. OME induced massive DNA damage, which consequently induced sustained autophagy followed by the activation of apoptosis and both events, led to cell death. *Also*, through a yet to be elucidated mechanism, OME downregulates survivin which might have contributed in triggering abortive autophagy and apoptosis.

## Data Availability

All datasets generated for this study are included in the manuscript.

## Author Contributions

NB, HA, KA, NA, and AE performed cell viability and Western blots. AsA and YA performed cell cycle analysis and colony assay. AyA performed the HPLC-MS analysis. RI designed the project, analyzed the data, and wrote the manuscript. All authors reviewed the manuscript.

### Conflict of Interest Statement

The authors declare that the research was conducted in the absence of any commercial or financial relationships that could be construed as a potential conflict of interest.
